# Melanoma cells with acquired resistance to dabrafenib display changes in miRNA expression pattern and respond to this drug with an increase of invasiveness, which is abrogated by inhibition of NF-κB or the PI3K/mTOR signalling pathway

**DOI:** 10.1186/1479-5876-13-S1-P5

**Published:** 2015-01-15

**Authors:** Simona Caporali, Ester Alvino, Adriana Amaro, Pedro Miguel Lacal, Lauretta Levati, Maria Grazia Atzori, Gian Carlo Antonini Cappellini, Federica Ruffini, Enzo Bonmassar, Ulrich Pfeffer, Stefania D’Atri

**Affiliations:** 1 Laboratory of Molecular Oncology, Istituto Dermopatico dell’Immacolata-IRCCS, Rome, Italy; 2 Institute of Translational Pharmacology, National Council of Research, Rome, Italy; 3 Integrated Molecular Pathology, IRCCS-AUO San Martino, National Cancer Research Institute, Genoa, Italy; 4 Department of Oncology and Dermatological Oncology, Istituto Dermopatico dell’Immacolata-IRCCS, Rome, Italy

## Background

The therapeutic success of BRAF inhibitors (BRAFi) is limited by the emergence of drug resistance [1,2]. Although several mechanisms underlying acquired resistance to BRAFi have been identified [1,2], further studies are required to disclose their entire spectrum. Altered expression and/or function of microRNAs (miRNAs) is involved in tumor onset, progression and drug resistance [3]. Here, we determined miRNA expression profiles of melanoma cells sensitive or resistant to the BRAFi dabrafenib, and investigated the effect of this drug on their invasiveness. We also evaluated the consequences of inhibiting NF-κB or the PI3K/mTOR signalling pathway on the invasive capacity of dabrafenib-resistant cells.

## Materials and methods

The BRAF^V600E^ mutant A375 melanoma cell line, and its dabrafenib-resistant subline A375R, generated in our laboratory, were analyzed for the levels of total and phosphorylated ERK1/2 and AKT, by Western blotting, and for miRNA expression pattern by using Affymetrix miRNA 3.1 arrays. A375 and A375R cells were also exposed to dabrafenib (100 nM, 48 hours) and tested for: a) in vitro invasion of extracellular matrix (ECM), under basal conditions and in response to VEGF-A (Matrigel covered Boyden chambers); b) VEGF-A secretion (ELISA). Finally, in vitro ECM invasion by A375R cells treated with the NF-κB inhibitor NEMO-binding domain (NBD) peptide (50 μM, 48 hours) or the PI3K/mTOR inhibitor GSK-2126458 (20 nM, 48 hours), alone or in combination with 100 nM dabrafenib, was evaluated.

## Results

A375R cells showed higher levels of phospho-ERK and phospho-AKT as compared with A375 cells. Eighty-nine miRNAs were up-regulated and 47 miRNAs were down-regulated in the A375R cells with respect to A375 cells. Gene Ontology analysis of the putative target genes of the top-ten down-modulated miRNAs revealed “regulation of cell motion” and “regulation of cell migration” as being among the most significantly enriched terms. A375 and A375R cells differently responded to dabrafenib, which strongly inhibited invasiveness and VEGF-A secretion in A375 cells, whereas it stimulated these functions in A375R cells. Treatment of A375R cells with NBD-peptide or with GSK-2126458 inhibited spontaneous invasion of ECM. Moreover, both agents completely abrogated dabrafenib-induced stimulation of ECM invasion.

## Conclusions

Our data show that changes in miRNA expression occur in melanoma cells with acquired resistance to dabrafenib, and that invasiveness of these cells is enhanced in the presence of the drug. They also indicate that targeting NF-κB or the PI3K/mTOR pathway could be an efficient therapeutic strategy in patients who develop resistance to BRAFi
